# Item-saving assessment of self-care performance in children with developmental disabilities: A prospective caregiver-report computerized adaptive test

**DOI:** 10.1371/journal.pone.0193936

**Published:** 2018-03-21

**Authors:** Cheng-Te Chen, Yu-Lan Chen, Yu-Ching Lin, Ching-Lin Hsieh, Jeng-Yi Tzeng, Kuan-Lin Chen

**Affiliations:** 1 Department of Educational Psychology and Counseling, National Tsing Hua University, Hsinchu, Taiwan, R.O.C; 2 Department of Physical Medicine and Rehabilitation, Taipei Veterans General Hospital, Taipei City, Taiwan, R.O.C; 3 Department of Physical Medicine and Rehabilitation, National Cheng Kung University Hospital, College of Medicine, National Cheng Kung University, Tainan City, Taiwan, R.O.C; 4 Department of Physical Medicine and Rehabilitation, College of Medicine, National Cheng Kung University, Tainan City, Taiwan, R.O.C; 5 School of Occupational Therapy, College of Medicine, National Taiwan University, Taipei City, Taiwan, R.O.C; 6 Department of Physical Medicine and Rehabilitation, National Taiwan University Hospital, Taipei, Taiwan, R.O.C; 7 Department of Occupational Therapy, College of Medical and Health Science, Asia University, Taichung, Taiwan, R.O.C; 8 Institute of Learning Sciences and Technologies, National Tsing Hua University, Hsinchu, Taiwan, R.O.C; 9 Department of Occupational Therapy, College of Medicine, National Cheng Kung University, Tainan City, Taiwan, R.O.C; National Yang-Ming University, TAIWAN

## Abstract

**Objective:**

The purpose of this study was to construct a computerized adaptive test (CAT) for measuring self-care performance (the CAT-SC) in children with developmental disabilities (DD) aged from 6 months to 12 years in a content-inclusive, precise, and efficient fashion.

**Methods:**

The study was divided into 3 phases: (1) item bank development, (2) item testing, and (3) a simulation study to determine the stopping rules for the administration of the CAT-SC. A total of 215 caregivers of children with DD were interviewed with the 73-item CAT-SC item bank. An item response theory model was adopted for examining the construct validity to estimate item parameters after investigation of the unidimensionality, equality of slope parameters, item fitness, and differential item functioning (DIF). In the last phase, the reliability and concurrent validity of the CAT-SC were evaluated.

**Results:**

The final CAT-SC item bank contained 56 items. The stopping rules suggested were (a) reliability coefficient greater than 0.9 or (b) 14 items administered. The results of simulation also showed that 85% of the estimated self-care performance scores would reach a reliability higher than 0.9 with a mean test length of 8.5 items, and the mean reliability for the rest was 0.86. Administering the CAT-SC could reduce the number of items administered by 75% to 84%. In addition, self-care performances estimated by the CAT-SC and the full item bank were very similar to each other (Pearson *r* = 0.98).

**Conclusion:**

The newly developed CAT-SC can efficiently measure self-care performance in children with DD whose performances are comparable to those of TD children aged from 6 months to 12 years as precisely as the whole item bank. The item bank of the CAT-SC has good reliability and a unidimensional self-care construct, and the CAT can estimate self-care performance with less than 25% of the items in the item bank. Therefore, the CAT-SC could be useful for measuring self-care performance in children with DD in clinical and research settings.

## Introduction

Developmental disabilities (DD), a diverse group of chronic conditions due to mental or physical impairments, cause many difficulties in areas such as language, mobility, learning, and self-care [1]. For a child with DD, independent self-care is critical to successful participation in life, which is one of the ultimate goals of early intervention and rehabilitation. Clinicians and researchers commonly use self-care measures to assess and monitor self-care function. According to the International Classification of Functioning, Disability and Health (ICF) from the World Health Organization, a child’s functioning can be assessed in terms of capacity and performance. The construct of “capacity” refers to what a child can do in a standard environment, whereas the construct of “performance” is what a child actually does perform in his/her daily environment [2]. A child’s routine performance is not always equal to his/her capacity. Assessing performance provides a way of indicating how the environment in which the measurement takes place affects a child’s daily activities [3, 4]. Therefore, as regards self-care function, assessment of self-care performance is necessary for clinicians and researchers to gain insights into how a child performs self-care activities routinely in his or her day-to-day context for further intervention planning.

Several measures have been developed to assess self-care performance in children with DD, including the Pediatric Evaluation of Disability Inventory (PEDI) [5], Functional Independence Measure for children (Wee-FIM^™^) [6], Vineland Adaptive Behavior Scales (VABS) [7], and School Function Assessment (SFA) [8]. However, two main challenges exist in these current pediatric self-care measures in children with DD. First, the contents of current pediatric self-care measures do not cover the whole age range for children (from 6 months to 12 years) to fit all children from infancy and preschool to school age, which hampers the longitudinal evaluation of intervention effectiveness. Second, the majority of pediatric self-care measures are lengthy and time-consuming for comprehensive administration, except the Wee-FIM^™^ (e.g., the administration times to measure children’s self-care using the PEDI, SFA, VABS, and Wee-FIM^™^ are respectively about 20, 15, 15, and 5 minutes). Unlike the others, the Wee-FIM^™^ only has 8 self-care items, which can be quickly assessed as a frontier screening self-care assessment. Therefore, assessors always face a dilemma between assessment efficiency and detailed examination of a child’s self-care performance. Children with DD and their caregivers, clinicians, and researchers are burdened by the administration of lengthy measures to understand children’s self-care performance. In summary, the two fundamental issues above threaten the usefulness of these current pediatric self-care measures in clinical and research settings, where time is always limited.

Computerized adaptive tests (CATs) can overcome the previously mentioned challenges in current pediatric self-care measures in children with DD. A CAT is an adaptive test employing item response theory (IRT) and computer technology. A CAT, like an experienced clinician, uses only the most informative items based on the respondent’s responses, and directs items relevant to the child’s estimated level of self-care according to previous responses. This approach differs from that of traditional fixed-form measures, in which clinicians need to use all the items for every child, regardless of the child’s responses. Items skipped in the CAT but administered in the traditional fixed-form tests are either too easy or too hard for the child and thus provide little information. Since CAT tailors every assessment to every child, fewer items and less time (e.g., within 5 minutes) are used in assessment without compromising the precision and accuracy of the test results [9–11], which can reduce the burden on parents, clinicians, and children. Through selecting and administering the most appropriate items from the item bank, the adaptive testing, relative to traditional fixed-form tests, can solve the dilemma between assessment efficiency and measurement precision.

**Fig 1:**
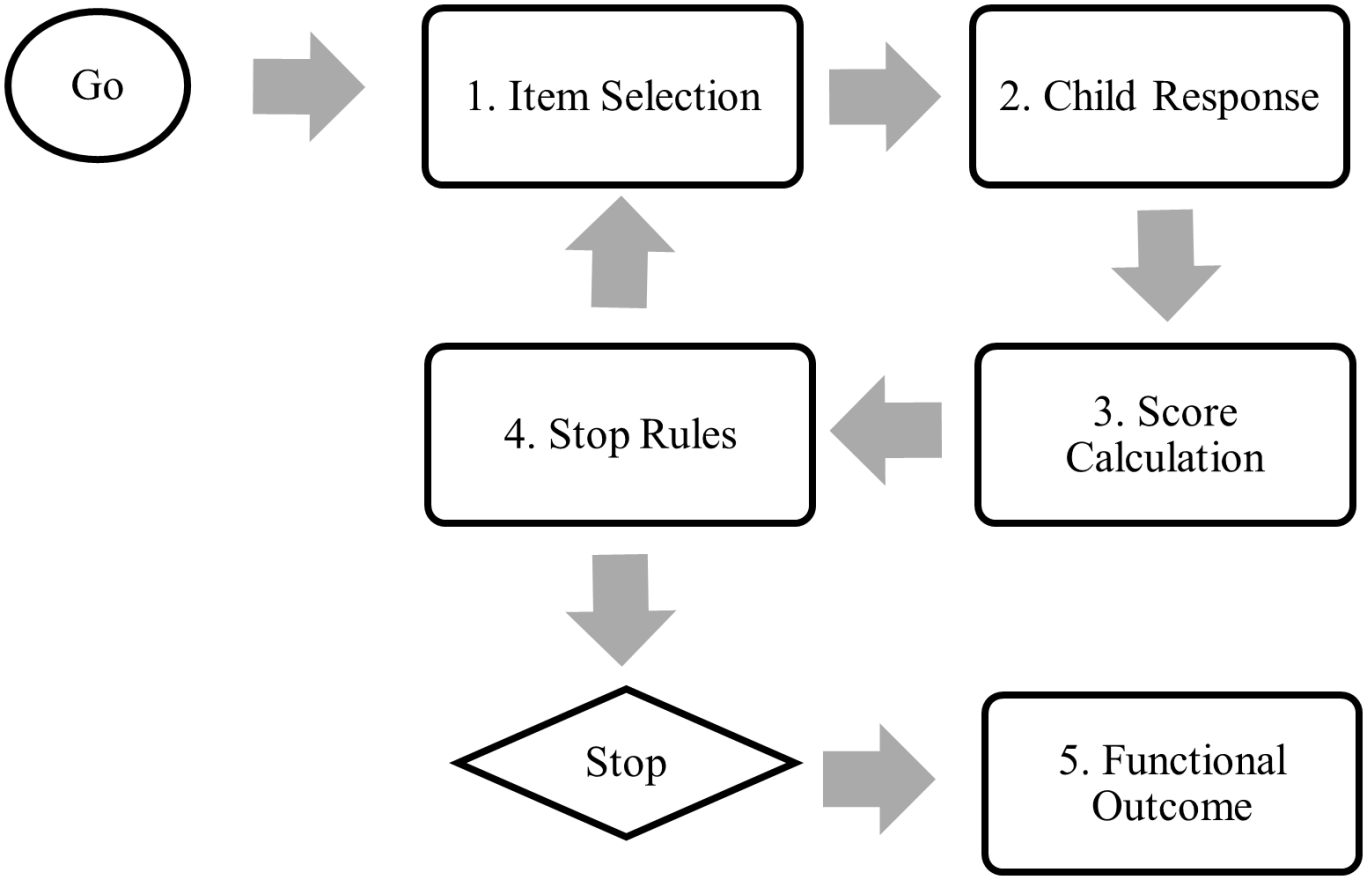
Illustration of the steps of computer adaptive testing (CAT).

The steps of a CAT in assessment are shown in Fig 1. For convenience, the self-care performance of children with DD is used as an example. In Step 1 (Item Selection), the CAT selects an initial item from the item bank to provide good discrimination of a child’s self-care. On the basis of the response(s) to previous item(s) (Step 2; Child Response), an initial score estimate and its reliability are calculated (Step 3; Score Calculation). In Step 4 (Stop Rules), the CAT checks the reliability and the test length (i.e., stopping rules) to decide whether to continue testing or not. If the CAT decides to continue, the CAT goes back to Step 1 and continues to select and test new items until either one of the stopping rules is satisfied. With the increased test length, the information about the child’s self-care performance is gathered efficiently, so the score estimates become more reliable. Once the stopping rule is satisfied, the CAT ends with a report of the estimate of the child’s self-care performance and its confidence interval (Step 5; Functional Outcome) [9,10].

Due to the need for efficient assessment, some CATs have been developed to measure children’s function, including the Pediatric Evaluation of Disability Inventory Computer Adaptive Test (PEDI-CAT) [12]. As regards self-care function, in the PEDI-CAT, the Daily Activities and Responsibility domains were respectively designed to measure children’s ability to carry out daily living skills and to manage life tasks that are important for the transition to adulthood and independent living. Therefore, the Daily Activities domain measures children’s capacity to execute self-care activities, but the Responsibility domain was not specifically designed for self-care and requires children to use functional skills assessed in the other three domains (i.e., Daily Activities, Mobility, and Social/Cognitive) to carry out life tasks (e.g., fixing a meal, planning and following a weekly schedule) [12]. Therefore, so far, there exists no pediatric CAT to specifically measure self-care function in daily life in children with DD based on the definition of performance and referencing the codes of the self-care domain of the ICF. In response to the lack of such a CAT, the purposes of this study were to develop an item bank, assess it for reliability and validity, and construct a CAT for measuring self-care performance (the CAT-SC) in children with DD aged from 6 months to 12 years in a content-inclusive, precise, and efficient fashion.

## Method

The study was divided into 3 phases: (1) item bank development, (2) item testing, and (3) a simulation study for determining the stopping rules of the CAT-SC and its performance. The study protocol was approved by three local institutional review boards: the Institutional Review Board of the National Cheng Kung University Hospital, the Taipei Veterans General Hospital Institutional Review Board, and the Chi Mei Hospital Institutional Review Board. All parents or guardians of the children provided written informed consent prior to data collection.

### Item bank development

We had two goals for the item bank: (1) the items in the item bank needed to inclusively cover the whole spectrum of self-care performance for children aged from 6 months to 12 years (both content- and age-inclusive); (2) the CAT-SC needed to be designed as a caregiver-reported measure. Therefore, the content of these self-care items should be easily observable by caregivers in daily life. The initial item bank was developed with the following resources: (1) self-care related theoretical frameworks: definition and scope of “self-care” of the International Classification of Functioning, Disability and Health-Children and Youth (ICF-CY) (World Health Organization, 2007) supplemented with a literature review; (2) current pediatric self-care measures; (3) expert review with a web-based platform constructed for experts to scrutinize the items; and (4) caregivers’ reports. In addition, for easier rating of children’s self-care performance, we developed the 4-point rating scale based on four resources: a literature review of the current pediatric self-care measures; expert review, with a web-based platform; caregivers’ reports; and our clinical experience.

The initial item bank had 47 items based on the ICF-CY (World Health Organization, 2007), the literature review, and current pediatric self-care assessments. We used a web-based platform constructed for experts to scrutinize the items and collect their comments. To improve the items in the item bank, our team performed content analysis and held several meetings to discuss the collected comments one by one. If needed, the experts were interviewed for clarification. In total, we consulted 37 experts in pediatrics (4 pediatricians or physiatrists, 6 professors, 23 occupational therapists, 3 physical therapists, and 1 speech therapist) for the items to be included. After adjustments based on expert review, the final bank of the CAT-SC grew to 73 items scored on a 4-point scale: 0—Total assistance, 1—Partial assistance, 2—Supervision, and 3—No assistance. The suggestions from expert review included (1) providing examples to make descriptions specific; (2) revising complex sentences; (3) dividing certain items into two to evaluate unique characteristics; (4) combining two items into one for comprehensive evaluation; and (5) deleting items with rare circumstances. We then conducted field tests on 10 caregivers to revise the wordings of the items where necessary. The final content of the 73 items was thus determined (S1 Table).

### Item testing

#### Participants

The 73 candidate items were administered to the caregivers of children with DD receiving rehabilitation at 3 teaching hospitals in Taiwan. Children were recruited if (1) they were aged between 6 months and 12 years; (2) they were formally provided with special medical, educational, or social services (i.e., children who were identified as needing pediatric rehabilitation by pediatricians or physiatrists based on traditional medical diagnostic categories, such as the International Classification of Diseases, 10th revision, clinical modification (ICD-10 codes) and Diagnostic and Statistical Manual of Mental Disorders, 4^th^ Edition (DSM-IV)); and (3) their caregivers gave informed consent. To ensure that the sample was both content- and age-inclusive to encompass the whole self-care spectrum in children with DD, two variables were considered in the sampling method: gender and age range. There were 17 age bands, each of which was expected to have 10 to 20 participants. In addition, the variable of level of self-care performance needed to be as various as possible. The variability of each stratum was monitored and analyzed during participant recruitment and data collection according to judgments based on clinical experience and statistical descriptive analysis.

#### Data analysis

To examine the construct validity, the unidimensional assumption of IRT was first examined to determine the correct number of latent factors, and items failing to meet the assumptions were deleted. Next, we compared the generalized partial credit model (GPCM) [13] and partial credit model (PCM) [14] to test for the invariance of item slopes. The GPCM allows an item specific slope that controls the discriminability of items along the latent construct of the self-care spectrum. After determination of a better fitted model, the item fit, reversal of step difficulties, and differential item functioning (DIF) were assessed. Misfit and DIF items were deleted and reversal categories were combined with adjacent ones. In the end, the item parameters were calibrated for the remaining items.

#### Evaluation of unidimensionality

In order to assess for the assumption of IRT and provide evidence of construct validity, the unidimensionality was examined using the Mplus software to perform confirmatory factor analysis (CFA) with a single factor structure. The estimation method for the CFA was the robust weighted least square estimator (WLSMV) [15] based on the tetrachoric and polychoric correlations of response data. Several fit indices, namely, the chi-square statistic, root mean square error of approximation (RMSEA), comparative fit index (CFI), and Tucker-Lewis index (TLI), were reported and checked for model-data fit. An insignificant chi-square statistic (with nominal level = .05), an RMSEA smaller than .07, and a CFI and a TLI greater than .95 indicated acceptable model-data fit. In addition, we removed from the item bank any items with factor loadings smaller than 0.4.

#### IRT model comparison

Item slope in the GPCM determines the discriminability around the latent trait that matches step difficulties, wherein higher discrimination provides more information and a smaller standard error of estimation. The likelihood ratio test was applied to examine whether item slopes were different from item to item by comparing the deviance of the two IRT models (i.e., GPCM and PCM). The data were first fit to the two models using PARSCALE 4.1 software [16], in which the deviance and number of parameters were reported. The difference of deviances was then calculated and compared to the chi-square distribution with the degree of freedom as the number of extra parameters used in the GPCM. A nominal level of .05 was used here; a significant difference in deviance indicates that the GPCM fits better and slopes are variant for items [16].

#### Fitting the IRT model

After model comparison, we fitted the preferred IRT model to the data. The goodness of fit for each item was assessed with PARSCALE’s *G*^2^ statistic, which is a likelihood ratio chi-square statistic. An item with significant *G*^2^ was considered misfitting and would be deleted or dealt with by merging response categories [16]. The step difficulties of each item were also examined for reversals. If the step difficulty parameters of the same item were not arranged in order, the response categories of that item were believed to be reversed. The reversed category was then combined with the adjacent category so that the remaining response scale could be deployed adequately.

Some of the items were suspected to be easier or more sensitive to a particular gender after controlling for ability, known as differential item functioning (DIF). The ordinal logistic regression method with purification was applied to assess both uniform and non-uniform DIF by using an R package named lordif [17]. Uniformity indicates that the effect is constant, which is similar to the differences in item difficulties, whereas non-uniformity indicates that the effect varies conditionally on the latent trait, which is similar to the differences in item discrimination. DIF items were deleted from the item bank to avoid test unfairness.

#### Simulating for stopping rules and validating the CAT

The catR package [18] under R was used to determine the stopping rules for the CAT-SC through a simulation study employing a real data set consisting of data from 215 participants, as adopted in the item bank testing. The relationship between the reliability and test length was investigated to determine the criteria for two stopping rules: maximum reliability and maximum test length. In the simulation study and construction of the CAT-SC, the maximum Fisher information (MFI) was used to select items, and the maximum *a posteriori* (MAP) method was used to estimate the latent self-care levels for participants because of its efficiency when applied in CAT.

The Pearson correlation coefficients of participants’ self-care estimates were derived from the CAT system under the previously determined stopping rules, and the entire item bank was considered as an index of concurrent validity for the CAT system.

## Results

A total of 215 caregivers of children with DD were interviewed with the 73-item CAT-SC item bank from May 2013 to April 2014 at 3 teaching hospitals in Taiwan. Table 1 shows the children’s and their caregivers’ characteristics, including the children’s diagnoses. The mean age of the children was 4.95 years (SD = 3.00 years), and boys (61.4%) outnumbered girls. The number of children in each age band ranged from 8 to 20, with a mean of 13 participants. With regard to caregivers, most caregivers were parents (89.8%), with more mothers (84.97%) than fathers. The mean age of the caregivers was 36.58 years (SD = 5.29 years). Most (60.5%) of the caregivers had at least 12 years of education and were currently married (92.6%).

**Table 1:** Descriptive statistics of the characteristics of children with developmental disabilities and their caregivers (N = 215).

Characteristics	Statistics
**Child characteristics**	
**Age (years): mean (SD), range**	4.95 (3.00), 0.50–11.50
**Gender (M/F): n (%)**	132 (61.4)/ 83 (38.6)
**Diagnosis: n (%)**	
**Non-specific developmental delay**	79 (36.7)
**Cerebral palsy**	46 (21.4)
**Autistic spectrum disorder**	21 (9.8)
**Genetic disorder**	16 (7.4)
**Preterm**	12 (5.6)
**Intellectual disability (without other diagnosis)**	8 (3.7)
**Others**	31 (15.3)
**Caregiver characteristics**	
**Caregiver’s age (years): mean (SD), range**	36.58 (5.29), 23–54
**Caregiver’s relationship with the child: n (%)**	
**Mother/ Father/ Others**	164 (76.3)/29 (13.5)/22 (10.2)
**Marital status: n (%)**	
**Married/ Others**	198 (92.6)/17 (7.4)
**Caregiver’s education: n (%)**	
**Primary school**	3 (1.4)
**Secondary school**	82 (38.1)
**University or above**	130 (60.5)

The average response rates from the categories “total assistance” to “no assistance” were .53, .15, .05, and .27, respectively. The third category (i.e., supervision) was seldom used by respondents, so the responses of the third category were combined with the fourth category in the following analysis to avoid redundancy. Meanwhile, 5 items probing female self-care ability, such as putting on a brassiere and menstrual care, were also deleted from the following analysis because of inadequate responses.

### Unidimensionality

The unidimensional assumption of IRT was met after the deletion of one item. Even though the chi-square statistic (*χ*^2^ = 3796.493, *df* = 2144, *p*-value<0.001) was significant due to the large sample size, the RMSEA (0.060), CFI (0.985), and TLI (0.985) showed good model fit. Meanwhile, the factor loadings were all above 0.4. The item (i.e., cleaning the ears) was removed because only 3 participants selected a response other than total assistance.

### IRT model comparison

The likelihood ratio test indicated that the GPCM fit the data better than the PCM (*χ*^2^ = 242.972, *df* = 66, *p*-value<0.001), which meant that item slopes were different from item to item. The estimates of item slopes ranged from 0.839 to 3.547, with a mean of 1.666 and a standard deviation of 0.57 for the final item bank. According to the likelihood ratio test and the variability of item slopes, the GPCM was chosen for the following analysis.

### Fitting the IRT model

After the GPCM was fitted to the responses of 67 items, one item was deleted from the item bank due to lack of fitness. Among the remaining 66 items, 41 items were either reversed or misfitted. The reason for item reversal might have been insufficient responses. The mean percentage of participants selecting the middle category of the reversed items was 8.68%, whereas the mean percentage of the non-reversed items was 23.74%. Considering the content validity and the number of reversed or misfitted items, these items were justified by combining the second response category (partial assistance) with the first one (total assistance). The final model-data fit was fairly good (*G*^2^ = 539.626, *df* = 601, *p*-value = 0.965). The subsequent DIF analysis showed that 10 items possessed either uniform or non-uniform gender DIF, so they were removed from the item bank. Examples of uniform DIF items are Washing hair, Putting on pants/skirts with a belt, Taking off pants/skirts that come with a belt, and Putting on socks, excluding fastening Velcro and shoe buckles as well as tying shoelaces, whereas examples of non-uniform DIF items are Putting on pants/skirts with buttons and zippers and Taking off pants/skirts, excluding unbuttoning and unzipping. Finally, the item bank consisted of 32 two-point items and 24 three-point items.

**Fig 2:**
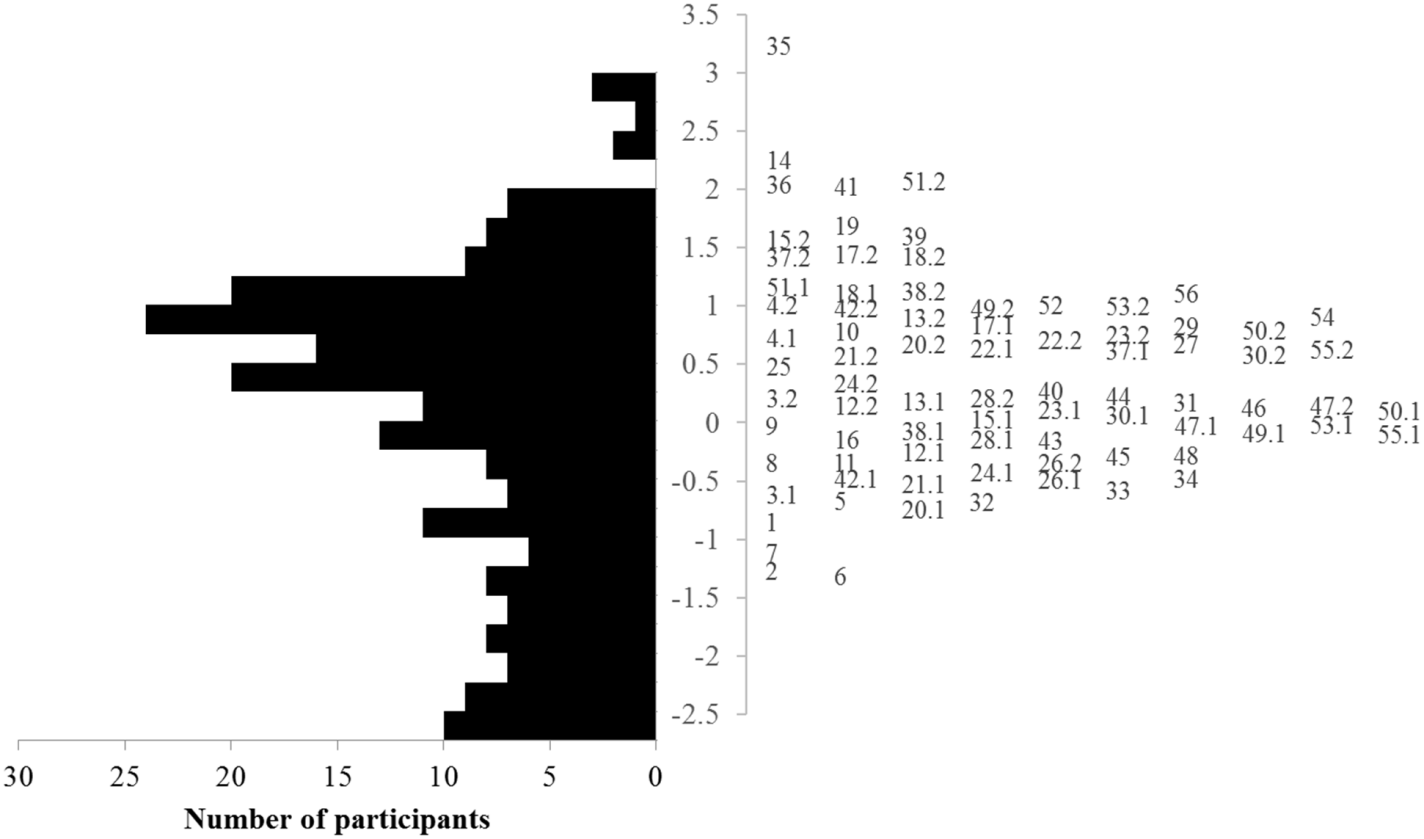
Item-person map of the final item bank. Note: On the left is the histogram of the estimated self-care performances from 215 children. On the right is the item difficulty aligned vertically. The number, for two-point items, indicates the item number, whereas the number also indicates the response category for three-point items. For example, 4.1 indicates the first step difficulty of item 4.

The item difficulty estimates ranged from -1.313 to 3.232. As compared to the standardized scale of children’s performances, the items were relatively difficult for our participants, as expected. Except for the most difficult 4 items, the standard error estimates of item difficulty were below 0.2, which means the difficulties were estimated quite accurately. Fig 2 presents the item-person map, which shows the histogram of participants’ self-care performance estimated from the final 56 items as well as the locations of item difficulty. Except for the participants with self-care performance lower than -1.5 logits, item and person had a good match.

### Stopping rules and validation of the CAT

**Fig 3:**
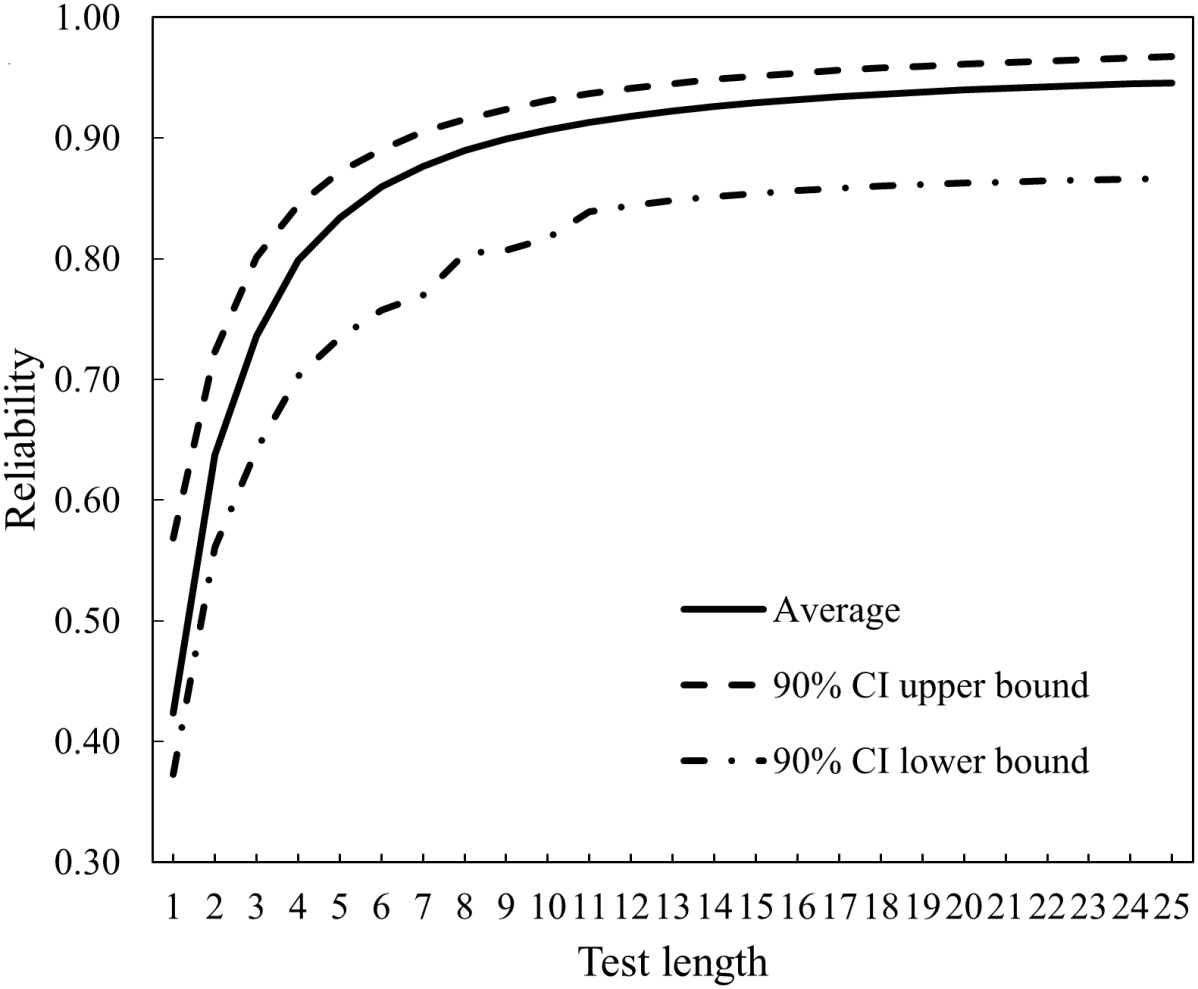
Reliability of CAT-SC under different test lengths. Note: CI = confidence interval.

The relationship between reliability, summarized as the mean and 90% confidence intervals of all respondents, and test length is shown in Fig 3. On average, the reliability was able to reach 0.9 after the administration of 9 items. After 14 items, the increase in reliability became gradual, and less than 5% of respondents with extreme self-care performance received a reliability smaller than 0.85. Therefore, the two stopping rules of the CAT-SC were a reliability of 0.9 or a test length of 14 items.

Since we set the initial self-care performance in the CAT at zero, the first item selected from the item bank would be the same for each respondent. The CAT-SC would estimate the respondent’s self-care performance as well as reliability and then select and administer the next item according to the revised self-care performance estimate. Once the reliability of self-care performance estimates reached 0.9 or the total test length hit 14 items, the CAT would stop and report (a) the final self-care performance estimate as well as its 95% confidence interval, (b) the estimation reliability, and (c) the age-equivalent value and the 95% confidence interval. Under the stopping rules of reliability (0.9) and test length (14 items), 85% of the 215 respondents received a reliability over 0.9, and the mean reliability of the remaining 15% of respondents was 0.86. Except for the respondents whose assessments were terminated by the test length rule (15%), the mean test length was 8.5, indicating that 84% of the items need not be administered. The correlation between self-care performance from the CAT and the entire item bank was 0.98. In summary, the two stopping rules worked as expected. As compared to administrating the entire item bank (56 items), administration of the CAT required 75% to 88% less items, which was almost the same across all age bands, yet the self-care performance estimated using the CAT was very close to the value derived from the entire item bank.

## Discussion

To the best of our knowledge, this is the first study to develop a CAT for assessing self-care performance based on the ICF. The results of this study show that the newly developed CAT-SC can efficiently measure self-care performance in children with DD whose performances are comparable to those of TD children aged from 6 months to 12 years as precisely as the whole item bank. The CAT-SC has good reliability and a unidimensional self-care construct, and it can reduce the number of items tested to less than 25% of that of the entire item bank. Therefore, the CAT-SC should be more efficient based on current technology for measuring self-care performance in children with DD. Moreover, the 56 Rasch-calibrated items in the final CAT-SC item bank have three clinical advantages. First, these unidimensional items can be used to represent the entire spectrum of children’s self-care performance. Second, the identified difficulties can be used as references for subsequent clinical decision making; for example, interventions can be targeted at the next difficulty level to improve the child’s self-care performance once the child’s current self-care performance has been identified. Last but not least, the ordinal scales of the raw sum scores can be transformed into the interval scale of logit scores, which can represent equal intervals between any two adjacent scores to quantify the self-care performance level of a child, the changes within a child, and differences between children.

This study developed a new item bank for the CAT-SC because our intention was to resolve the dilemma between assessment efficiency and detailed examination of children’s self-care performance inherent in the current pediatric self-care measures. Based on a careful development process with theoretical grounds, expert validation, and field testing, the CAT-SC item bank covers the spectrum of self-care performances for children with 6 months to 12 years old, and these self-care items can be easily observable by caregivers in daily contexts. In addition, these items have also been examined with strict psychometric analysis. Therefore, the CAT-SC has scientific robustness and clinical appropriateness.

From the item-person-map, the locations of children and items matched fairly well. Only one item (item number 35) was located too high to find any child who had corresponding self-care performance. The reason is that the targeted population, children with DD, always need a certain amount of assistance with daily self-care activities and are not totally independent. Moreover, about 19% of the children (N = 41) with locations lower than -1.5 logits did not have corresponding items to evaluate their self-care performance properly. To improve the utility of the CAT-SC, future studies could recruit more children who have better function in the higher end of the self-care performance spectrum to better estimate the difficulty of items, or such studies could add simple items through expert interview, participant interview, or the combination of other self-care tools to better estimate the abilities of children with lower self-care performance.

In the analysis, some gender-specific items were removed due to insufficient numbers of respondents. However, in order to enhance the content validity, more responses to these items should be collected. In fact, these items were also specifically administrated after the adaptive testing process of the CAT-SC. Whether or not to include these items in the item bank will also be examined by fitting to the GPCM once the amount of responses is sufficiently large. In the future, a gender-specific self-care score might be also reported by the CAT-SC.

In the item bank development, the rating for each item originally had four response categories, based on a literature review and expert consultation. Even though these categories were collapsed to either two or three categories due to the low selection rates of certain categories or to conform to the IRT model, all four response categories are still provided in the CAT-SC to respondents so as to collect more information to prove the usefulness of that response category when more respondents are recruited. This use of more response categories will allow the collection of more information about children’s self-care performance as well as additional clinical meaning. On the other hand, parents should be interviewed to confirm whether certain response categories (e.g., partial assistance or supervision) were truly redundant for the reversed items.

The satisfactory evidence of this study is only applicable to children with DD and cannot be generalized to typically-developing children. For clinical applicability, the CAT-SC was originally designed for children with DD, but it was also intended to cover the entire spectrum of typically-developing children from 6 months to 12 years of age in the development of item bank. According to the good psychometric evidence of the CAT-SC in children with DD, the utility of the CAT-SC in typically-developing children can be explored. The CAT-SC is believed to have the potential for use as a quick online screening assessment tool for identifying both DD and typically-developing children who are in need of intervention on their self-care performance.

The efficiency of the CAT-SC should be evaluated with real data in the future in addition to the previously-identified issues regarding improving the item bank (i.e., adding simpler items and enhancing the reliability and validity by recruiting more children with DD). Even though we have reported how much the number of items administered can be reduced by administrating CAT, the actual time spent in responding to the paper and pencil version and that used for completing the CAT should be tested to confirm that the assessment time has actually been reduced and that the CAT-SC provides efficiency without sacrificing precision or accuracy.

## Conclusions

The CAT-SC was developed to provide accurate and efficient assessment of this important self-care domain utilizing IRT item calibrations for clinical use in pediatric children with diverse DD. In this study, we tested its item bank and the CAT algorithm to provide evidence of its psychometric properties and its utility in children with DD. In conclusion, newly developed CAT-SC can efficiently measure self-care performance in children with DD whose performances are comparable to those of TD children aged from 6 months to 12 years as precisely as the whole item bank. The item bank of the CAT-SC has good reliability and a unidimensional self-care construct, and the CAT procedure can reduce the testing items by more than 75%. Therefore, the CAT-SC should be useful in clinical and research settings.

## Supporting information

S1 Table Items for the computerized adaptive test assessing self-care performance of children with developmental disabilities. (DOCX)

S1 Dataset Raw responses of the 73-item self-care item bank. (SAV)
