# Supplementary material for: Item-saving assessment of self-care performance in children with developmental disabilities: A prospective caregiver-report computerized adaptive test
Source: PLoS One. 2018 Mar 21;13(3):e0193936. doi: 10.1371/journal.pone.0193936 (PMC5862472; doi:10.1371/journal.pone.0193936)
Supplement: S1 Table — (DOCX) [file pone.0193936.s001.docx]

**S1 Table. Question Bank for A Computer Test Assessing Self-Care Performance of Children with Developmental Disabilities**

Note: The original version is in Chinese, and this English version has not gone through an entire standard translation process of forward-backward translation, expert validation, and psychometric evaluation. The English version we currently provide may not be suitable for academic or clinical uses for English users.

| **Item**  **Number** | **Item** | **Explanations** |
| --- | --- | --- |
| **d550** | | |
| 1 | Indicating need for eating | When the child is hungry, he/she can indicate his/her need for eating. |
| 2 | Eat food with hands | For example, eat cookies with hands |
| 3 | Eating with Spoons | Finish a meal by eating with a spoon with little food dropped on the table. The assessment focus of this item includes the performance of the child using a spoon to finish a meal. If the child can use a spoon but is unable to finish a meal independently due to other reasons (such as distraction or dependence), the rating should be based on the level of assistance that the child needs regularly. |
| 4 | Eating with Chopsticks | Finish a meal by eating with chopsticks with little food dropped on the table. The assessment focus of this item includes the performance of the child using chopsticks to finish a meal. If the child can use chopsticks but is unable to finish a meal independently due to other reasons (such as distraction or dependence), the rating should be based on the level of assistance that the child needs regularly. |
| **d560** | | |
| 5 | Indicating need for drinking | When feeling thirsty, the child can indicate his/her need for drinking. |
| 6 | Drinking – Drinking from a container with a lid, excluding the action of opening the lid | For example, drink from nipple bottles, baby bottles with handles or safe locks, or other cups or mugs with a straw or a lid. This item does not include the action of opening the lid but emphasizes on whether the child can have a simple control of the liquid. |
| 7 | Drinking—Using straws | For example, drink with straws. |
| 8 | Drinking – Drinking from a container without a lid | For example, drink from a container without a lid, such as a mug. This item emphasizes on the child’s good control of the liquid without spilling. |
| 9 | Drinking – Drink from a container with a lid, including the action of opening the lid | For example, open the lid of a container, such as a plastic bottle or a mug with a lid, and drink from it. |
| 10 | Take water to drink | Pour water from a jug, a water bottle, or a kettle into a cup, or drink water from drinking fountains. |
| **d510** | | |
| 11 | Washing Hands – Water | Wash hands and rinse with water. |
| 12 | Washing Hands – Hand Sanitizer | Use hand sanitizers, such as soap bars or hand soaps, to wash hands and rinse with water. |
| 13 | Washing Face – Wiping Face | Use a towel or a handkerchief to clean and wipe face. |
| 14 | Washing Face – Facial Cleanser | Use soap bars or facial cleaners to wash face and rinse with water. |
| 15 | Washing Head | Use shampoo or other hair cleansing products to wash hair and rinse with water. |
| 16 | Taking a Shower | Use soap bars or body wash to take a shower and rinse with water. |
| 17 | Drying Hands | Use a towel, a handkerchief, a paper towel, or a hand dryer to dry hands. |
| 18 | Drying Body | Use a towel or a bath towel to dry one’s body. |
| 19 | Drying Hair | Use a towel to dry hair until the hair is not dripping wet. |
| 20 | Blow-drying Hair | Use a hair dryer to blow-dry hair. |
| **d540** | | |
| 21 | Putting on pullover tops, excluding buttoning or zipping up | For examples, put on t-shirts without considering the action of buttoning or zipping up. |
| 22 | Putting on front-open tops, excluding buttoning or zipping up | For examples, put on jackets or shirts without considering the action of buttoning and zipping up. |
| 23 | Putting on tops with buttons | For example, button up the shirt after putting it on. |
| 24 | Putting on tops with zippers | For example, zip up the jacket after putting it on. |
| 25 | Putting on female’s bra | Include the steps of putting on, clasp, and adjust the bra. |
| 26 | Putting on pants/skirts, excluding buttoning and zipping up | For example, put on pants/skirts with elastic band |
| 27 | Putting on pants/skirts with buttons and zippers | For example, close the button on the pant/skirt or zip up after putting it on. Exclude the steps of fastening the belt. |
| 28 | Putting on pants/skirts with a belt | For example, fasten the belt after putting on the pant. |
| 29 | Taking off pullover tops, excluding closing the buttons and the zipper | For example, take off t-shirts with no need to unbutton and unzip. |
| 30 | Taking off front-open tops, excluding unbuttoning and unzipping | For example, take off jackets or shirts with no need to unbutton and unzip. |
| 31 | Taking off tops with buttons | For example, take off shirts after unbuttoning. |
| 32 | Taking off tops with zippers | For example, take off jackets after unzipping. |
| 33 | Taking off female’s bra | Include the steps of unclasping and taking off the bra. |
| 34 | Taking off pants/skirts, excluding unbuttoning and unzipping | For example, take off pants/skirts with elastic bands. |
| 35 | Taking off pants/skirts with buttons or zippers | For example, take off pants/skirts after unbuttoning and unzipping. The steps of unfastening the belt. |
| 36 | Taking off pants/skirts that come with a belt | For example, take off pants/skirts after unfastening the belt. |
| 37 | Putting on socks | For example, put on ankle socks. |
| 38 | Putting on socks, excluding fastening Velcro and shoe buckles as well as tying shoelaces | For example, wear slippers, slides, or shoes with loose Velcro and shoelaces. |
| 39 | Putting on shoes with Velcro | For example, fasten Velcro or shoe buckles after putting on shoes. |
| 40 | Putting on shoes with shoelaces | For example, tie shoelaces after putting on shoes. |
| 41 | Taking off socks | For example, take off ankle socks. |
| 42 | Taking off shoes, excluding unfastening Velcro and shoe buckles as well as untying shoelaces | For example, take off slides or shoes with loose Velcro or shoelaces. |
| 43 | Taking off shoes with Velcro/shoe buckles | For example, take off shoes after unfastening Velcro or shoe buckles. |
| 44 | Taking off shoe with shoelaces | For example, take off shoes after untying shoelaces. |
| 45 | Choosing appropriate clothing | Choose appropriate clothing according to weather, occasions, and culture. For example, wear short sleeves in summer, long sleeves in winter, uniform for school, and so on. |
| **d520** | | |
| 46 | Skin care | For example, after checking skin, put lotion, sunscreen lotion, baby powder, or ointment on the skin. |
| 47 | Brushing teeth | Include the steps of squeeze toothpaste, using a toothbrush to brush teeth, and gargle. |
| 48 | Cleaning teeth | Use dental floss or toothpicks to clean teeth. |
| 49 | Combing hair | Use a comb to comb hair, excluding tying the hair or doing hair styling |
| 50 | Tying hair | Use a comb to comb hair, and tie or style the hair. |
| 51 | Cutting fingernails | Use nail clippers or scissors to cut nails of both hands. |
| 52 | Cutting toenails | Use nail clippers or scissors to cut nails on both feet. |
| 53 | Cleaning the nose | Use toilet paper or handkerchief to blow and wipe one’s nose. |
| 54 | Cleaning the ears | Use cotton swabs or ear picks to clean ears. |
| **d530** | | |
| 55 | Regulating urination – day | When feeling the urge of urinating, the child can control the urge without wetting the pant. This item emphasizes the child’s performance of controlling his/her urination during the day without going to the restroom or using the toilet or potty. |
| 56 | Regulating urination –night | When sleeping at night, the child can control the urge of urinating without wetting the pant. This item emphasizes the child’s performance of controlling his/her urination during the day without going to the restroom or using the toilet or potty. |
| 57 | Indicating need for urination | When feeling the urge of urinating, the child knows how to express his/her need for urination. |
| 58 | Using toilet/potty | Include setting the toilet seat (such as putting down the toilet seat), peeing or stooling in the toilet or potty, and flush the toilet or potty. |
| 59 | Using toilet for urination on his/her own | Include the steps of setting the toilet seat (such as putting down the toilet seat), taking off the pant/skirt, peeing in the toilet or potty, flushing the toilet or potty, and putting on the pant/skirt. |
| 60 | Regulating defecation | The child will not soil on his/her paint during the day and night. This item emphasize the child’s performance of regulating defecation without going to the restroom, using the toilet/potty, or wiping his/her bottom. |
| 61 | Indicating need for defecation | When feeling the urge of defecating, the child can express his/her need for defecation. |
| 62 | Using the toilet for defecation on his/her own | Include the steps of setting the toilet seat (such as putting down the to let seat), taking off the pant/skirt, stooling in the toilet or potty, wiping the bottom, flushing the toilet or potty, and putting on the pant/skirt. |
| 63 | Predicting menstrual cycle | For example, the child can predict menstrual cycle and prepare or bring feminine care products, such as sanitary napkins or tampons. |
| 64 | Cleaning during menstrual cycle | During the menstruation, the child knows how to use sanitary napkins, tampons, or other feminine care products to keep herself clean. For example, replace sanitary napkins or tampons, and wipe to clean the skin. |
| **d570** | | |
| 65 | Ensuring one’s physical comfort | The child is aware of the changes in the environment and takes appropriate action to make his/her body comfortable without feeling too hot, too cold, or too wet and with sufficient light. For example, when the child feels too hot or too cold, he/she knows to take off or put on clothes or turn on or off the fan or air conditioner. Turn on the light when it is dim. |
| 66 | Keeping healthy diet | Understand the importance of keeping a healthy diet and be aware of his/her daily diet. Choose nutritious food to keep healthy. For example, the child can understand the importance of keeping a balanced diet, so when it is time for him/her to choose his/her meals, he/she can choose food that is good for health without being picky and choosing junk food as his/her main meals. |
| 67 | Physical fitness | Understand the importance of exercising to health. Be aware of personal physical activity to keep oneself healthy. For example, the child has the habit of exercising or keeping a certain amount of exercise regularly. The child can also finish daily routine or school activity energetically without feeling exhausted and even has spare energy to enjoy leisure activities and deal with sudden events. |
| 68 | Managing medications and following health advice | Take medicine by following doctors’ instructions when sick, and take doctors’ advice on diet or lifestyle. |
| 69 | Seeking advice or assistance from caregivers or professionals | When being ill, the child will seek advice or assistance from caregivers or professionals. |
| 70 | Treating minor wounds | Understand the principles of treating wounds. The child can handle minor mosquito bites, abrasions, or cuts. For example, be able to handle minor abrasions due to a fall while running and cuts by a utility knife by performing a simple hemostasis, cleaning, applying medication, and bandaging (such as using adhesive band-aids). |
| 71 | Avoid infectious  diseases | Have a preliminary understanding of infectious diseases, such as paying attention to cleanliness, avoiding eating at random, washing hands frequently, and reducing public access during epidemic diseases. |
| **d571** | | |
| 72 | Looking after one’s safety | Be aware of and pay special attention to dangerous objects or the environment in order to avoid getting hurt. For example, avoid very hot pots, do not play on busy roads, and be cautious when using sharp objects and playing nearby the stairs. |
| 73 | Responding to emergencies | Know how to seek help in an emergency. For example, know to call 110 or 119 when getting hurt or lost, contacting people who can provide help, or ask adults in the vicinity for help. |
